# Comment on: Inflammatory indexes in predicting postoperative atrial fibrillation

**DOI:** 10.1590/1806-9282.20250999

**Published:** 2026-01-09

**Authors:** Cihan Aydın, Aykut Demirkıran, Hüseyin Orta

**Affiliations:** 1 Tekirdağ Namık Kemal University, Faculty of Medicine, Department of Cardiology – Tekirdağ, Turkey.

Dear Editor,

We read the article by Erkan et al. entitled "The role of the systemic inflammatory response index in predicting postoperative atrial fibrillation" with great interest^1^. We congratulate the authors for their valuable contribution. We would like to make some additional comments. Postoperative atrial fibrillation (POAF) is a common complication after cardiac surgery, which causes increased morbidity and extended hospitalizations^2^. Inflammation is recognized as a crucial factor in the pathogenesis of POAF that occurs following coronary artery bypass grafting. This inflammatory reaction increases the risk of atrial fibrillation by aggravating myocardial injury and disrupting electrical stability. In this study, the value of biomarkers reflecting the degree of inflammation and immune response, such as Systemic Immuno-Inflammatory Index (SII) and Systemic Inflammatory Response Index (SIRI), in predicting POAF was investigated. Although SIRI and SII values were higher in the patient group developing POAF, no significant difference was observed (p>0.05). In addition, age and family history of coronary artery disease were found to be important factors significantly affecting the development of POAF (p=0.048 and p=0.01, respectively).

The reason for the similarity of the inflammation indices may be the intensive use of acetylsalicylic acid, which has an anti-inflammatory effect, in both groups. In addition, the small number of patients included in the study and the calculation of these indices from blood only once at a certain time may have prevented accurate results.

Preoperative measurement of left atrium diameters, volumes, and volume indices associated with atrial fibrillation (AF) in these patient groups could have contributed to the study^3,4^.

The use of antiarrhythmic drugs such as amiodarone, which will reduce the incidence of AF in the preoperative period, may affect the development of POAF. This situation could have been stated by the authors in the article. In addition to the SIRI and SII, other indices such as the neutrophil–lymphocyte ratio and Prognostic Nutritional Index could have been examined in the study. The receiver operating characteristic curve analysis could have been performed to calculate the cut-off values for predicting POAF for these indices.

The authors recognize that the study's retrospective, single-center design restricts its generalizability.

## Data Availability

The datasets generated and/or analyzed during the current study are available from the corresponding author upon reasonable request.
